# ﻿*Miconiagaragoana* - Melastomataceae: A new rheophytic species from the eastern Andes of Colombia

**DOI:** 10.3897/phytokeys.247.119563

**Published:** 2024-10-11

**Authors:** Humberto Mendoza-Cifuentes, William Ariza-Cortés, Lyndon Carvajal Rojas

**Affiliations:** 1 Jardín Botánico de Bogotá José Celestino Mutis, Cl. 63 #68-95, Bogotá DC., Colombia Jardín Botánico de Bogotá José Celestino Mutis Bogotá Colombia; 2 Universidad Distrital Francisco José de Caldas, Herbario Forestal UDBC. Carrera 3 # 26A-40, Bogotá, Colombia Universidad Distrital Francisco José de Caldas Bogotá Colombia

**Keywords:** Eastern Mountains, endemism, Miconieae, northern Andes, rheophytic plants, riparian environments, Ambientes riparios, Cordillera Oriental, endemismo, Miconieae, norte de los Andes, plantas reofíticas

## Abstract

A new species, *Miconiagaragoana*, from riparian environments of the northern Cordillera Oriental of Colombia, is described. This is the second species exclusive to rheophytic conditions that grows in the Andean forest in Colombia and is characterized by the presence of stellate-lepidote trichomes on young structures and inflorescences, terminal inflorescences, 4-merous (rarely 5-merous) flowers, oblong-subulate anthers with an apical pore and 2-locular ovary. The differences with other rheophytic species occurring in Colombia are noted and it is argued that it is related to other Andean species with bluish-green ripe fruits such as *M.squamulosa* and *M.symplocoidea*. This new species is so far known only from Colombia and its threat category is suggested as Critically Endangered” (CR).

## Abstract (es)

Se describe una nueva especie, *Miconiagaragoana*, de ambientes riparios del norte de la Cordillera Oriental de Colombia. Esta es la segunda especie exclusiva de condición reofitica que crece en bosques andinos de Colombia y se caracteriza por la presencia de cubiertas de tricomas estrellado-lepidotos en las estructuras jóvenes e inflorescencias, inflorescencias terminales, flores 4-meras /raras veces 5-meras), anteras oblongo-subuladas con un poro apical y ovario 2-locular. Se establecen las diferencias con otras especies reofíticas presentes en Colombia y se argumenta que está relacionada con especies de andinas de frutos maduros color verde-azuloso como *M.squamulosa* y *M.symplocoidea*. Esta nueva especie hasta ahora se conoce sólo de Colombia y se sugiere su categoría de amenaza como En Peligro Crítico (CR).

## ﻿Introduction

The genus *Miconia* Ruiz & Pav. is one of the largest in the Melastomataceae family and among the largest exclusive to the Neotropical region (Goldenberg et al. 2008). Recently, phylogenetic analyses (Michelangeli et al. 2016, 2022) redefined this genus to include all Neotropical Melastomataceae species characterized by fleshy fruits, small flowers, and multiflorous inflorescences. Previously, these species were grouped under the tribe Miconiae, which included 18–20 genera exclusively distributed in the Neotropics. The genus comprises an estimated 1900 species, with about 600 found in Colombia (Almeda et al. 2016; Mendoza Cifuentes et al. 2021; Michelangeli et al. 2022).

*Miconia* species grow in variety of environments at all altitudes, but are particularly rare along rivers and streams, especially in lowland areas of tropical rainforests. For example, *Miconiaaplostachya* (Bonpl.) DC. and *M.riparia* Triana are found along blackwater rivers and streams in Amazonia and Antioquia department in Colombia, while *M.rheophytica* Posada-Herrera & Almeda is the only specie restricted to rocky margins of torrential rivers in the Andes of Colombia (Hoyos-Gómez and Bernal 2018; Posada-Herrera and Almeda 2018).

In order to document the floristic diversity of Sub-Andean forest remnants in Colombia, numerous expeditions have been carried out in the departments of Boyacá and Santander, with the discovery of a new species of *Miconia* from riparian environments. This manuscript describes this new species, exclusively associated with stream margins at elevations near 2000 meters above sea level. The habitat characteristics of the species are outlined, and an assessment of its extinction threat is provided.

## ﻿Material and methods

This new species was discovered through a comprehensive review of collections housed in regional herbaria, coupled with field expeditions conducted in previously unexplored regions of the Eastern Cordillera in Colombia. Several collections were located in the herbaria UDBC and JBB. Measurements of vegetative parts were made in dry herbarium material using a digital caliper with a precision of 0.1 mm. Measurements of the floral parts and fruits were based on fresh flowers preserved in alcohol from the plants designated as types. Trichome types follow Wurdack (1986). Field photos were compiled and a map of the occurrence of this new taxon in Colombia was generated using Arc-GIS version 10.2.1. Preliminary conservation status was assessed by estimating the Area of Occupancy with GeoCat (http://geocat.kew.org/editor) and applying the IUCN Red List Category criteria (IUCN 2012, 2017).

## ﻿Results

### ﻿Taxonomic treatment

#### ﻿ Miconia garagoana

Taxon classificationPlantaeMyrtalesMelastomataceae

Humberto Mend., Ariza-Cortés & L.Carvajal sp. nov.

1C276159-98E0-5E4C-9DC6-E620FE8CF477

urn:lsid:ipni.org:names:77349957-1

Figs 1 , 2

##### Diagnosis.

Rheophytic shrubs with stellate-lepidote trichomes in distal branches and inflorescences; leaves linear-elliptic, 3-nerved or 3- slightly plinerved, length-to-width ratio is greater than 5.5:1; terminal inflorescences with 1-9(-14) flowers, flowers 4-merous (rarely 5-merous), anthers oblong-subulate with a ventrally oriented pore, ovary 2-locular, fruits bluish-green with large and relatively few sedes. Similar to *Miconiariparia* Triana but differs in the indumentum of stellate-lepidote trichomes (vs. indumentum of pinoid trichomes in *M.riparia*), flowers predominantly 4-merous (vs. 5-merous). Also similar to *Miconiarheophytica* Posada-Herrera & Almeda, but the latter has branches with dense indumentum of dendritic ferruginous trichomes, ciliate leaf margins, dichasial axillary inflorescences, 5-merous flowers and 3-locular ovary.

**Figure 1.**
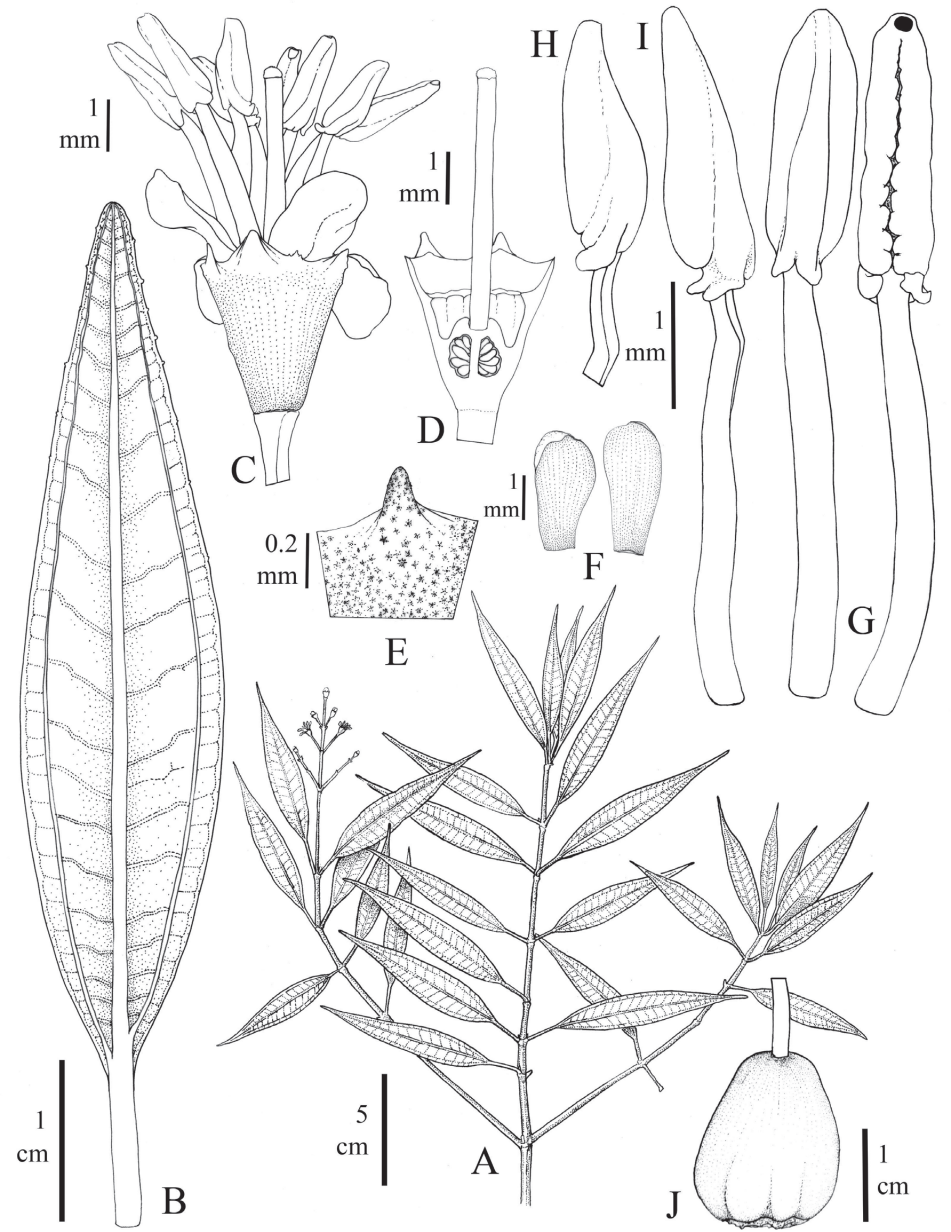
*Miconiagaragoana* Humberto Mend., W.Ariza & L.Carvajal **A** flowering branch **B** leaf seen from the underside **C** flower in lateral view **D** longitudinal cut of the ovary-hypanthium **E** dorsal tooth of calyx **F** petals **G, H, I** stamens in different views **J** ripe friut. Illustration based on W. Ariza-Cortés et al. 4855 (UDBC).

##### Type.

Colombia. • Boyacá: Municipio de Garagoa, vereda Ciénega Valvanera, Reserva Privada El Secreto, en borde de Quebrada; 2100 m elev.; 5°7'29"N, 73°16'42"W; 12 Apr 2016 (fl); *W. Ariza-Cortés et al. 4855* (holotype: UDBC!; isotypes: JBB!, COL! [Branches with inflorescences and fruits were deposited in the supplementary spirit collection -Anthoteca UDBC]).

**Figure 2.**
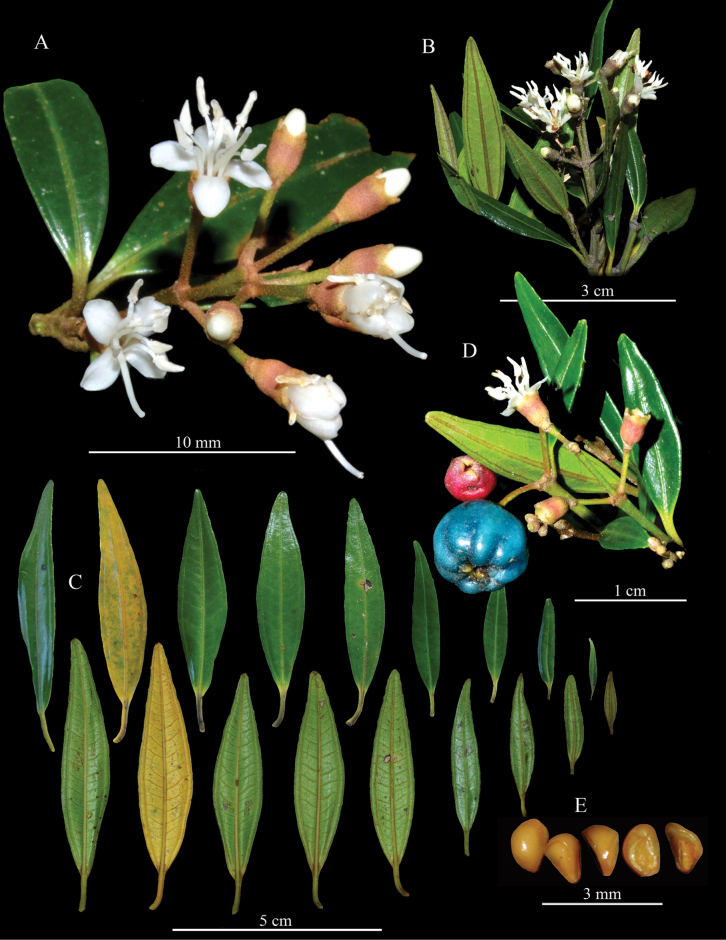
*Miconiagaragoana* Humberto Mend., W.Ariza & L.Carvajal **A, B** different views of flowering branch **C** leaf variation **D** branch with flowers and fruits **E** seeds. All photos by Lindon Carvajal and William Ariza.

##### Description.

***Shrub*** 30–250 cm tall, rheophytic; foliage dense; external bark smooth and whitish; primary branches sympodial, plagiotropic; young branches quadrangular, flattened and slightly channeled, older branches becoming oblong-terete and exfoliating in thin flakes; apical buds with dense indument of stellate-lepidote trichomes (type 38 of Wurdack 1986); ***internodes*** 0.9–3.5 cm long, 1.7–3.3 mm wide. ***Leaves*** decussate, isophyllous; ***petiole*** 5–17 mm long, slightly channeled adaxially, glabrous; ***blade*** 40–85 × 7–17 mm, linear-elliptic, apex and base acute, margin entire, revolute and denticulate toward apex, adaxial surface green, shiny (fresh material) and glabrous, in mature leaves yellow, abaxial surface clear pale green, with sparse stellate-lepidote trichomes especially along the middle vein; ***venation*** with one pair of secondary veins accompanying the middle vein, nerved or slightly plinerved to 1–3 mm, 22–34 pairs of tertiary veins lateral to the middle vein, 1–3 mm apart in the middle, central vein raised on both surfaces, tertiary veins blurred on the lower side. ***Inflorescence*** 2.5–5 cm long, paniculate, sparsely branched, terminal, sessile or with a peduncle 5–20 mm long, with a sparse to dense indument of stellate-lepidote trichomes (type 38); 1-9(-14) flowers, sessiles; central axis with 1–5 branching nodes; branch apices regularly with one flower; basal nodes with linear bracts 2–5 mm long, distal nodes with linear-subulate bracts 1.7–4.4 mm long; flower supported by two linear-triangular bracteoles 0.7–1 mm long. ***Flowers*** 4-merous, rarely 5-merous, diplostemonous. ***Hypanthium*** 2.3–2.4 × 1.7–2 mm, obconical, externally with dense indument of stellate-lepidote trichomes (type 39) ca. 0.09 mm diameter; internally glabrous and slightly ribbed; free thalamus of ovary ca. 0.9 mm long. ***Calyx*** lobed, externally with indument similar to the hypanthium; tube ca. 0.2 mm long; lobes 0.2–0.3 × 1.2–1.3 mm, wide triangular; dorsal teeth 0.55–0.6 mm long, triangular and exceeding the lobe length. ***Corolla*** patent; petals 2.9–3.2 × 1.4–1.7 mm, spatulate, apex rounded, white, glabrous. ***Stamens*** isomorphic, glabrous, white; filaments 3.3–3.7 mm long; anthers 1.8–2.1 × 0.4 × 0.6 mm, oblong-subulate, with a ventrally oriented pore ca. 0.2 mm diameter; basal connective with two little ventral lobules 0.2–0.35 mm long. ***Ovary*** 1.6–1.7 mm long, 2-locular, basal part fused to the hypanthium ca. 1 mm long, apical part free of the hypanthium ca. 0.6 mm long, rounded, glabrous; style 5–6.1 mm long, cylindric; stigma 0.3–0.4 mm diameter, punctiform. ***Fruit*** 15–18 × 8–15 mm, pyriform, slightly ribbed, bluish-green when ripe, with 12–18 seeds. ***Seeds*** 1.3–2 × 1.2–1.5 mm, ovoid and angled, antiraphal portion symmetrical and ovate; testa smooth, shiny, light yellow.

##### Phenology.

In Santander, flowering was recorded in November, while in Boyacá flowering was observed between February and July. Fruiting occurred between April and August. At least in the Boyacá locality, active flowering and fruiting events are presumed to occur for most of the year.

##### Habitat and distribution.

**Figure 3.**
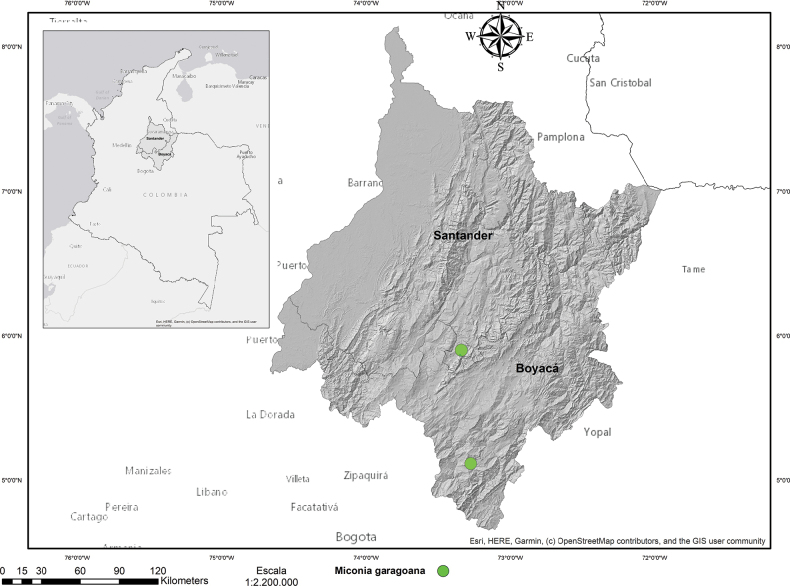
Distribution of *Miconiagaragoana* Humberto Mend., W.Ariza & L.Carvajal in northern South America.

**Figure 4.**
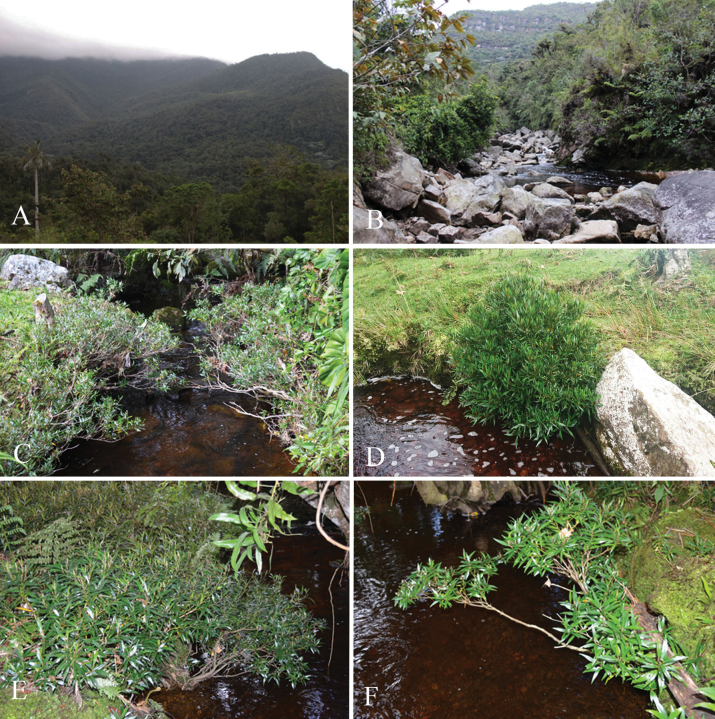
*Miconiagaragoana* Humberto Mend., W. Ariza & L. Carvajal **A** Andean forests in the area of origin **B** habitat **C–F** different views of growth habit. All photos by Lindon Carvajal and William Ariza.

*Miconiagaragoana* is endemic to Colombia in the North of the Eastern Cordillera. This species has been recorded in the departments of Boyacá and Santander, within relatively undisturbed Andean forests at altitudes ranging from 2000 to 2200 meters above sea level (Fig. 3). It is a rheophytic plant, which grows exclusively along the banks of streams and rivers, typically on slopes characterized by moderate to steep inclinations (Fig. 4).

##### Etymology.

The specific epithet refers to the municipality of Garagoa in the department of Boyacá, where the majority of individuals of the species have been observed.

##### Conservation status.

*M.garagoana* is found in remnants of riparian vegetation along rivers and streams in Andean humid forests, typically within a matrix of pastures designated for livestock grazing (Fig. 4). Considering its limited area of occupancy AOO = 8 km^2^, recorded only in two locations, it is proposed as Critically Endangered (CR), according to criteria B2a of IUCN (2012, 2017).

##### Specimens examined (Paratypes).

**Colombia** • **Santander**: Municipio de Gambita, Cueva de Choco, creciendo en borde de quebrada en el interior de la caverna; 2176 m elev.; 05°54'7.17"N, 72°20'22.73"W; 01 Nov 2018 (fl); H. Mendoza 21578 (JBB, UDBC).

## ﻿Discussion

*Miconiagaragoana* exhibits distinctive morphological characteristics. It is recognized by the rheophytic condition and its abundant branching, occasionally featuring plagiotropic main branches that incline under the influence of water currents. This species displays an indumentum of stellate-lepidote trichomes on its distal branches, inflorescences, and the underside of its leaves. The linear-elliptic leaf is a distinguishing feature commonly found in species inhabiting the banks of rivers and streams, representing an adaptation to withstand the constant impact of water currents (van Steenis 1987). Furthermore, other remarkable morphological traits of *Miconiagaragoana* include terminal inflorescences with few flowers (1–14), predominantly 4-merous flowers, oblong-subulate anthers featuring a ventrally oriented pore, and a 2-locular ovary. In addition, this species is characterized by its mature fruits, which feature an bluish-green color, and its large and relatively few seeds (less than 20).

The most vegetative similar species to *Miconiagaragoana* is *M.riparia*, also present in Colombia, but the latter has dense rufous indumentum of pinoid trichomes on stems (vs. stellate-lepidotes in *M.garagoana*), and 5-merous flowers (vs. predominantly 4-merous) and dark purple or black fruits (vs. bluish-green). It is also similar to *Miconiarheophytica*, but the latter has branches with dense rufous indumentum of dendritic trichomes, ciliate leaf margins, dichasial axillary inflorescences, 5-merous flowers, yellow anthers, 3-locular ovary, globose to subglobose fruit with a bright indigo blue colour, and seeds ovoid with lateral and antiraphal symmetrical planes elliptic to elliptic-ovate (Posada-Herrera and Almeda 2018). Additionally, *Miconiariparia* and *M.rheophytica* grow below 1000 m, while *M.garagoana* grows around 2100 m elevation.

According to the number of petals, *M.garagoana* could be associated with the Ulmarioides complex (recently revised by Tiernan and Michelangeli (2018). However, all species of this complex always present 4-locular ovary and pink or fuchsia connective of the anther, so there is not full certainty.

Perhaps the species most closely related to *Miconiagaragoana* are *M.squamulosa* (Sm.) Triana, and *M.symplocoidea* Triana, considering that they share the indumentum of stellate-lepidote thrichomes in vegetative parts and flowers, the shape of the stamens, and especially the bluish-green ripe fruits with angled ovoid seeds with smooth testa. *Miconiasquamulosa* is part of section Cremanium, while *M.symplocoidea* is part of section Miconia (Goldenberg et al. 2013). However, these sections proposed by Cogniaux (1891) are considered artificial (Goldenberg et al. 2008).

The rheophytic condition is one of the distinctive characters of *Miconiagaragoana*. Rheophyte is a term coined by van Steenis in 1987 to describe a biological group of flood-tolerant plants that are confined to the beds of swift-running streams and rivers in nature, growing there up to flood level, but not beyond the reach of regularly occurring flash floods (van Steenis 1987; Santana Costa et al. 2020).

In Melastomataceae, about 23 rheophytic species are reported, of which six, including the present novelty, correspond to the genus *Miconia* (Hoyos-Gómez and Bernal 2018; Posada-Herrera and Almeda 2018; Santana Costa et al. 2020). Among these species are *Miconialinearis* (Gleason) Michelang, *M.mulleola* Wurdack, *M.rheophytica*, *Miconiariparia* and *Miconiasalicina* (Ser. ex DC.) Mabb. However, there are gaps in the information on the hábitat of the species, so it is not known with certainty how many, and which, species are restricted to this rheophytic condition. On the other hand, some of the species on record as being rheophytic are not exclusive to riparian environment, as is the case with *Miconiamulleola*. In order to guide conservation plans for riparian environments, the addition of this type of information would be very useful.

## Supplementary Material

XML Treatment for Miconia garagoana

## ﻿Additional information

### Author ORCIDs

Humberto Mendoza-Cifuentes https://orcid.org/0000-0002-5685-9338

William Ariza-Cortés https://orcid.org/0000-0002-8423-8256

Lyndon Carvajal Rojas https://orcid.org/0009-0003-4065-8296

### Data availability

All of the data that support the findings of this study are available in the main text.
